# The pathogenesis, diagnosis, and management of metastatic tumors to the ovary: a comprehensive review

**DOI:** 10.1007/s10585-017-9856-8

**Published:** 2017-07-20

**Authors:** Ondřej Kubeček, Jan Laco, Jiří Špaček, Jiří Petera, Jindřich Kopecký, Alena Kubečková, Stanislav Filip

**Affiliations:** 1 Department of Oncology and Radiotherapy, Faculty of Medicine and University Hospital in Hradec Králové, Charles University, Sokolská 581, 500 05 Hradec Králové, Czech Republic; 2 Department of Obstetrics and Gynecology, Faculty of Medicine and University Hospital in Hradec Králové, Charles University, Hradec Králové, Czech Republic; 3 The Fingerland Department of Pathology, Faculty of Medicine and University Hospital in Hradec Králové, Charles University, Hradec Králové, Czech Republic

**Keywords:** Ovary, Neoplasm, Tumor, Ovarian cancer, Krukenberg tumor, Metastasis

## Abstract

Secondary tumors of the ovary account for 10–25% of all ovarian malignancies. The most common tumors that give rise to ovarian metastases include breast, colorectal, endometrial, stomach, and appendix cancer. The correct diagnosis of secondary ovarian tumors may be challenging as they are not infrequently misdiagnosed as primary ovarian cancer, particularly in the case of mucinous adenocarcinomas. The distinction from the latter is essential, as it requires different treatment. Immunohistochemistry plays an important role in distinguishing primary ovarian tumors from extra-ovarian metastases and, furthermore, may suggest the primary tumor site. Despite extensive study, some cases remain equivocal even after assessing a broad spectrum of antigens. Therefore, gene expression profiling represents an approach able to further discriminate equivocal findings, and one that has been proven effective in determining the origin of cancer of unknown primary site. The available data concerning secondary ovarian tumors is rather limited owing to the relative heterogeneity of this group and the practical absence of any prospective trials. However, several intriguing questions are encountered in daily practice, including rational diagnostic workup, the role of cytoreductive surgery, and consequent adjuvant chemotherapy. This review seeks to address these issues comprehensively and summarize current knowledge on the epidemiology, pathogenesis, and management of secondary ovarian tumors, including further discussion on the different pathways of metastatisation, metastatic organotropism, and their possible molecular mechanisms.

## Introduction

The tendency of various tumors to form ovarian metastases has been known for a long time. In 1896, German gynecologist and pathologist Friedrich Ernst Krukenberg described a supposedly new kind of primary ovarian cancer which he named as “fibrosarcoma ovarii mucocellulare carcinomatodes” [1]. The metastatic nature of this tumor was revealed 5 years later by Kraus, who was probably the first to use the eponym “Krukenberg tumor” [2]. Krukenberg tumors (KT) are secondary ovarian tumors histopathologically defined as carcinomas that have a significant component (arbitrarily defined as >10% of the tumor) of mucin-filled signet-ring cells [3–5]. However, this definition is not followed by all authors and the designation KT is sometimes applied loosely to all metastatic tumors to the ovary, which should be avoided [5]. The most common origin of KT is from gastric cancer (in up to 70% of cases), especially the poorly cohesive/signet ring-cell type [6]. Even though KT are probably the best-known secondary tumors of the ovary (STOs), they account for only about 30–40% of all secondary ovarian tumors [7]. Metastases from other sites that do not comply with the histopathological definition of KT are frequently found in ovaries, including colon, breast, small intestine, and pancreatic cancer, malignant melanoma, and others [8]. It is not rare that the detection of STOs precedes the diagnosis of the primary tumor, which may be small and asymptomatic at the time of diagnosis. This puts forth a great challenge for both clinicians and pathologists in making the correct diagnosis, essential for adequate treatment. Although the prognosis of STOs is generally poor, there are differences across various primary tumors [9]. Furthermore, metastasectomy may improve the prognosis in select cases [10].

## Epidemiology

STOs account for 10–25% of all ovarian malignancies [11–14]. The most common tumor types metastasizing to ovaries include breast, colorectal, endometrial, stomach, and appendix cancer (Table 1) [15]. The available data regarding tumor incidence varies greatly across different studies, which could be explained by factors such as geographic distribution, age of patients, laboratory methods used in diagnostics, and the experience of the pathologist examining the samples [12]. Both the incidence of STOs and the proportion of distinct primary tumors giving rise to STOs display a marked variability across different geographical regions; e.g. Asian countries have been consistently reporting higher rates of STOs compared to European countries (Table 1), which can be explained by a higher prevalence of tumors metastasizing to ovaries in this region [11, 16]. While gastric cancer accounts for 23.4 and 30.4% of STOs in Japan and Korea, respectively, it can be found in only 4.5% of STOs patients in Netherlands, reflecting its incidence in these countries [11, 15, 17]. Conversely, breast cancer and CRC are the most common primary tumors metastasizing to ovaries in Europe and USA [15, 18].

**Table 1:** Review of retrospective studies on STO

	Alvarado-Cabrero et al. [13]	Bruls et al. [14]	Demopoulos et al. [18]	de Waal et al. [15]	Kondi-Pafiti et al. [12]	Lee et al. [11]	Moore et al. [8]*	Skírnis-dóttir et al. [19]*	Yada-Hashimoto et al. [17]
Geographical region	Mexico	Netherlands	USA	Netherlands	Greece	Korea	USA	Sweden	Japan
Total cases of malignant ovarian tumors [n]	950	9748	553	764	520	821	718	10,955	304
Secondary ovarian tumors [n]	150	2312	96	116	97	112	59	255	64
Percentage of STO [%]	15,7	23,7	17,4	15,2	18,7	13,6	8,2	2,3	21,1
Median age at diagnosis of STO [years]	51_(av.)_	59	54,8 _(av.)_	49,5	55	46,8 _(av.)_	55	56	50,3 _(av.)_
Premenopausal [%]	n/a	n/a	42,7	44,0	44,3	65,2	n/a	n/a	n/a
Postmenopausal [%]	n/a	n/a	57,3	39,7	55,7	34,8	n/a	n/a	n/a
Primary tumor detected first [%]	44,0	n/a	59,4	82,8	90,7	42,9	n/a	56,9	n/a
Primary tumor and STO detected simultaneously [%]	35,3	n/a	30,2	9,2	n/a	54,5	n/a	n/a	n/a
STO detected first [%]	20,6	n/a	10,4	8,0	n/a	2,6	n/a	n/a	n/a
STO in the left ovary [%]	n/a	19,8	25	n/a	22,7	n/a	20,3	n/a	n/a
STO in the right ovary [%]	n/a	26,7	31,3	n/a	24,7	n/a	13,6	n/a	n/a
STO in both ovaries [%]	n/a	46,3	43,8	69,0	52,6	54,5	66,1	n/a	n/a
Krukenberg tumors [%]	23,3	n/a	n/a	8,6	20,6	n/a	n/a	n/a	17,2
Primary tumor site (nonGYN)									
Breast	13	14,3	33,3	27,6	15,5	1,8	8,5	29,4	14,1
Large intestine	30	33,2	12,5	19,8	15,5	41,2	32,2	29,4	10,9
Stomach	16	4,5	6,3	6,0	24,7	30,4	6,8	16,1	23,4
Small intestine	–	1,6	2,1	2,6	1,0	–	6,8	2,7	–
Appendix	13	7,3	1,0	1,7	3,1	1,8	20,3	3,1	1,6
Pancreas	12	1,0	1,0	0,9	2,1	–	5,1	2,7	–
Biliary tract	15	0,6	1,0	–	–	3,6	1,7	1,2	1,6
Liver	4	–	–	–	–	–	–	0,4	–
Melanoma	–	–	–	2,6	1,0	–	–	1,6	–
Sarcoma	–	–	–	1,7	1,0	–	–	–	3,1
Lung	–	0,8	1,0	–	1,0	1,8	–	2,0	1,6
Others	–	7,0	3,1	3,4	1,0	1,8	3,4	4,0	4,7
Unknown origin	–	15,1	0	7,8	–	8,0	16,9	7,5	1,6
Primary tumors site (GYN)									
Endometrium	23	17,1	14,6	19,8	22,7	8,9	–	–	20,3
Cervix	4	1,1	2,1	5,2	9,3	0,9	–	–	14,1
Fallopian tube	–	–	1,0	0,9	3,1	–	–	–	3,1
Contralateral ovary	–	–	20,8	–	–	–	–	–	–

*av*. average

*Only non-gynecological tumors included

The age at diagnosis of STOs seems to be associated with the origin of the primary tumor. Patients with primary tumors localized within the gastrointestinal (GI) tract are generally older than those with a primary tumor localized outside the GI tract. Conversely, patients with breast cancer metastasizing to ovaries are significantly younger than those with primary tumors located elsewhere than the breast [15]. Patients with STOs are generally younger than those with primary epithelial ovarian cancer [15]; among them, patients with KT represent the youngest subgroup with an average age of 45 years at the time of diagnosis [6]. The younger age at STO diagnosis can be attributed to the fact that primary tumors metastasizing to ovaries arise at a younger age than primary ovarian tumors [5]. Furthermore, greater ovary vascularization in young women facilitates hematogenous spread [20].

## Prognosis

Patients with STOs have a generally poor prognosis, as it usually represents an advanced stage of the disease. The survival of patients with STOs is significantly worse than those with primary ovarian cancer (5-year survival rate of 18.5 vs. 40.0%) [19], although it is worth noting that there are differences in prognosis depending on the primary tumor [9]. The prognosis of patients with STOs originating from the genital tract is significantly better than of those with tumors originating outside the genital tract (median overall survival [OS] is 48 vs. 12 months) [12]; whereas STOs originating from pancreatic and small intestine cancer have the poorest prognosis [15]. Moreover, the prognosis of STOs that originate from a previously diagnosed malignancy is considerably better than in tumors presenting primarily as ovarian metastases. Several prognostic factors have been identified: pre-operative carbohydrate antigen 125 (CA 125) level in serum, age at diagnosis of STOs, pre-operative STO size [21], primary tumor origin [9], presence of peritoneal dissemination [20], extent of cytoreductive surgery [16, 22], and unilaterality versus bilaterality [21]. Furthermore, mutations of the *SMAD family member 4* (*SMAD4*) and *lysine methyltransferase 2D* (*KMT2D*) genes are associated with reduced OS in ovarian metastases from CRC [23], indicating that somatic mutation profiling may provide additional prognostic information.

## Pathogenesis

The exact mechanism of how extra-ovarian tumors give rise to ovarian metastases remains unclear. Several mechanisms have been proposed, including lymphogenous, hematogenous, and transcoelomic pathways [24]. The former two mechanisms involve the spreading of tumor cells via lymphatic and blood vessels, respectively, whereas transcoelomic dissemination means the spreading of the tumor cells across the peritoneal cavity [25]. Furthermore, it seems that different tumors do metastasize through different pathways; for example, a hematogenous spread seems to be the most frequent pathway in colon cancer [8]. This observation is further supported by immunohistochemical analyses revealing a higher rate of vascular invasion (67%) and a lower rate of lymphangio-invasion (0%) in ovarian metastases from colorectal cancer compared to gastric cancer [26]. Conversely, a retrograde lymphogenous spread seems to be involved in gastric cancer metastases [26]. There are several possible explanations. First, a rich mucosal and submucosal lymphatic plexus in the stomach enables lymphogenous metastases even at early stages of the disease [27]. The anatomy and evolution of the lymphatic system provides another explanation [26]. The urogenital lymph vessel tract gives rise to the receptaculum chyli via the lumbar trunks. The receptaculum chyli joins intestinal trunks, which connect via celiac lymph nodes to the gastric, hepatic, pancreaticolienal, and mesenteric (superior mesenteric and mesocolic) nodes. The short distance from the receptaculum chyli to the gastric nodes enables the gastric cancer cells to metastasize easily via the receptaculum chyli to the urogenital lymph vessel trunks, which supply the ovaries [26]. Moreover, tumor infiltration of retroperitoneal lymphatic nodes by gastrointestinal (GI) cancers may lead to lymphatic vessel obstruction and subsequently countercurrent of the lymph flow into the ovaries [28]. Transcoelomic dissemination, which is the main pathway of metastasis in primary ovarian cancer [25], does not seem to play a major role in STO development. There are no signs of peritoneal dissemination in most cases of STOs and the capsular surface of affected ovaries is usually smooth, without tumor deposits [6]. Notably, it seems that the metastatic routes may combine in some cases, especially in advanced GI tumors [26].

### Metastatic organotropism and its role in the development of STOs

The propensity of tumors to metastasize predominantly to specific secondary sites has been known for over a century and is referred to as metastatic organotropism [29]. The underlying molecular mechanisms for this phenomenon are rather complex and remain largely unexplored. Regardless, several mechanisms have been found, including the attraction of tumor cells to specific organs, adhesion of cells to the endothelium in a specific organ, survival in the metastatic site, angiogenesis at the metastatic site, the pre-metastatic niche, and the ability of tumor cells to extravasate into a specific organ [29]. Furthermore, recent findings suggest that the loss of specific miRNAs may be involved in organotropism, providing a selection advantage for cancer cells in a specific organ [30, 31]. Generally, the principle of organotropism can be understood as a crosstalk between intrinsic molecular properties of the primary tumor cells and the specific features of the metastatic site microenvironment [32]. A combination of specific molecular markers possesses a strong ability to predict a potential metastatic site [29, 33]. Several molecular patterns associated with metastatisation to the brain, lungs, and bones have been identified in breast and lung cancer [32]; however, no markers specific for metastatisation to ovaries have been identified yet. A retrospective study including 38 cases of ovarian metastases from CRC used next-generation somatic mutation profiling to assess 341 cancer-associated genes [23], finding an increased number of mutations in the *KRAS, SMAD4*, and *NTRK1* genes in ovarian metastases, compared to a cohort of 543 cases without ovarian metastases. However, whether presence of these mutations is responsible for ovarian organotropism remains unclear. Another study compared somatic mutation profiles in 26 primary CRCs and matched ovarian metastases [34], and while the known driver genes were mostly concordant, the primary CRCs showed a considerably higher number of passenger mutations than corresponding ovarian metastases. This leads to the hypothesis that a large number of sub-clones are present within the primary tumor, out of which only certain sub-clones possess the ability of homing into ovaries [34]. Owing to a limited number of samples, neither of these studies achieved the identification of mutation profiles specific for ovarian metastases.

Taking into account that the majority of high-grade serous ovarian cancers are considered to originate in the fimbria of the fallopian tube and subsequently metastasize into ovaries [35], it can be hypothesized that high-grade serous ovarian carcinomas and STOs might share some molecular patterns related to ovarian organotropism. The effort to identify specific molecular patterns associated with preferential metastasizing to ovaries is warranted, as they might aid in treatment decision-making (e.g. prophylactic bilateral ovarectomy if these patterns are found within the primary tumor).

## Clinical signs

Similar to primary ovarian cancer, STOs remain asymptomatic until the tumor grows into a certain size [21]. This is the reason why most patients attend the clinic with advanced disease. Presenting symptoms are non-specific and can be found in a majority of patients (70%) upon diagnosis [21]; these include abdominal pain (42%), postmenopausal bleeding (18%), weight loss (6%), and an increasing abdominal girth (15%) [8]. Vaginal bleeding and change in menstrual habits can be attributed, at least in some cases, to luteinization of the ovarian stroma and consequent hormonal production [4]. These changes are induced by STOs and may lead, although rarely, to hirsutism and virilization [36]. Ascites is present in 39% of cases at diagnosis, which is less common than in primary ovarian cancer [37]. Patients with STOs that originate from the appendix may present with pseudomyxoma peritonei [8]. Generally, there are no clear differences between the symptoms arising from primary and secondary ovarian malignancies [37].

## Diagnosis

Making the correct diagnosis is the most challenging step in the management of STOs, as they frequently mimic primary ovarian cancer [38]. Moreover, the detection of STOs precedes diagnosis of the primary tumor in up to 40% of cases, especially in patients with colon and stomach cancer [9, 39]. The ability to make a clear distinction between the primary ovarian tumor and STOs is essential as each requires different treatment [15]. The diagnostic process should comprise thorough physical evaluation, basic blood and biochemical analyses, imaging methods, and endoscopy. The most frequent primary tumors causing STOs in a particular geographical region should be considered when searching for the primary tumor. The only reliable method to distinguish STOs from primary ovarian tumors comes through histopathological examination, preferably utilizing immunohistochemistry (IHC). Unfortunately, despite thorough diagnostic evaluation, the primary tumor remains unknown in about 15% of cases [14].

### Imaging methods

The role of imaging methods is to provide information on the extent of the disease and to identify a potential primary tumor site. A computed tomography (CT) scan of the chest, abdomen, and pelvis, with the use of intravenous contrast material, should be performed as a baseline evaluation, as this is considered a standard in cancer of an unknown primary site (CUP) [40–42]. None of the routinely used imaging methods, including CT, ultrasonographic (US), and magnetic resonance (MR) imaging has proved reliable in distinguishing primary ovarian cancer from STOs, although primary ovarian tumors tend to be multilocular more frequently than STOs on US and MR images [43, 44]. Also, 2-[fluorine-18]-fluoro-2-deoxy-d-glucose positron emission tomography integrated with computed tomography (^18^F-FDG PET/CT) is unable to distinguish STOs from primary ovarian tumors [45]. Despite the superior sensitivity of PET/CT over CT, there are several limitations, including the inability to detect smaller lesions (<1.0 cm) and tumors with low or no FDG uptake (e.g. renal cell carcinoma, breast carcinoma, and some GI cancers) [46, 47]. At present, there is limited available data from prospective trials to address the differences in the diagnostic value of CT and PET/CT in detecting the primary tumor site of CUP. Møller et al. found that PET/CT does not represent a clear diagnostic advantage over CT alone in patients with multiple metastases of an unknown primary site [48]. Therefore, PET/CT cannot be recommended as a routine imaging method in patients with STOs. However, it might be useful in select patients when considering local or regional therapy [40].

### The role of CA 125 and other tumor markers in STO management

Elevated CA 125 can be found in 80% of women with primary epithelial ovarian cancer and in 70% of those with STOs [15, 49]. However, the pre-operative level of serum CA 125 does not differ between primary and secondary ovarian tumors [39], and the sensitivity of CA 125 in detecting STOs is significantly lower than in primary ovarian cancer [21]; therefore, CA 125 does not seem a useful biomarker in the primary diagnostics of STOs. The CA 125/CEA ratio, however, might be of clinical use in distinguishing primary ovarian tumors from colorectal carcinoma (CRC) metastases [50]. Additionally, the combination of a complex ovarian mass with papillary projections presenting on the US and serum CA 125 level above 170 U/mL shows a high positive predictive value (95.7%) for primary ovarian cancer [37]. Additional available data suggests that elevated pre-operative CA 125 and CA 19-9 levels might be associated with an adverse prognosis [21, 22]. The use of other tumor markers in the diagnostic process is limited owing to their non-specificity [51]; several tumor markers can be elevated in a non-specific way, providing no diagnostic, predictive, or prognostic value [52]. Furthermore, there are no current prospective clinical trials to clarify the role of epithelial tumor markers in STOs. Taken together, the routine use of epithelial tumor markers in the primary diagnosis of STOs cannot be recommended. Tumor markers, however, may provide useful information regarding the treatment response [40].

### Endoscopic methods

The routine use of endoscopic methods is generally not recommended for patients with CUP unless there are specific symptoms, imaging, or pathological abnormalities suggesting GI tract to be the primary tumor site [40]. However, considering that the majority of STOs are of a GI origin, endoscopic investigation seems to be a reasonable approach. It also provides a non-invasive method to obtain a histopathological specimen. It is the authors’ opinion that, in countries with a high incidence of gastric and colorectal cancer, the endoscopic evaluation (esophagogastroduodenoscopy and/or colonoscopy) should always be considered as a frontline diagnostic tool in STOs of an unknown primary site, unless the histopathological investigation rules out a possible GI origin.

## Histopathological diagnosis

### Gross morphology

Metastases to ovaries are generally smaller than primary ovarian carcinomas and usually contain cysts [15], most STOs are smaller than 10 cm in diameter [12]. Notably, metastases from breast cancer are usually smaller, while metastases from the GI tract (especially from colon cancer) tend to be bigger and may resemble primary ovarian tumors [12, 15]. Bilateralism is more frequent in STOs than in primary ovarian cancer, with both ovaries affected in up to 69% cases [15]. The tendency toward metastasizing into both ovaries is even more obvious in KT, which are bilateral in more than 80% of cases [6]. While tumor metastases from breast, stomach, and appendix carcinomas are mostly bilateral, the metastases from colorectal carcinoma tend to be unilateral [14].

The combination of tumor size and bilateralism proved to be of potential clinical use to distinguish primary ovarian tumors from STOs when assessed intra-operatively [53]. Although not specific, this algorithm might provide additional information to the intra-operative frozen section histology at the time when the definitive histological examination is not yet available [53]. Furthermore, additional data suggests that bilateralism may be associated with an adverse prognosis [9, 21].

In general, the gross features favoring metastases include small size (<10–12 cm), bilateralism, a nodular growth pattern, and the presence of a tumor on the surface and/or in the superficial cortex of the ovary [54].

### Histology

The histology of STOs is usually in correspondence with that of the primary tumor, with mucinous adenocarcinoma being the most common histological finding [12]. A poorly cohesive/signet-ring cell morphology can be observed in the vast majority of metastases from gastric cancer [6]. The predominant histological type of breast cancer metastasizing to ovaries is invasive ductal carcinoma, followed by invasive lobular carcinoma, with the latter not uncommonly manifesting as KT [12, 15]. Metastatic endometrial malignancies are mainly adenocarcinomas, while squamous cell carcinomas are the most common histological type of cervical metastases [15]. Other rarer histological types include metastatic sarcoma, melanoma, and lung cancer [12]. Metastatic adenocarcinomas with mucinous features represent a diagnostic issue, as they might be misdiagnosed as primary ovarian neoplasms [38]. It was reported that as many as 45% of metastatic ovarian tumors of colon origin initially were misdiagnosed as primary ovarian cancers [55]. A prominent tubular component and luteinization of the ovarian stroma can be found in some KT, leading to confusion with Sertoli cell or Sertoli-Leydig cell tumors; however, Sertoli and Sertoli-Leydig cell tumors are rarely bilateral, and signet-ring cells are usually absent [4]. Previous history of malignancy may raise suspicion of STOs, but the metastatic nature of any ovarian mass always must be considered as it may be the first finding in previously undiagnosed extra-ovarian cancer. An intra-operative frozen section biopsy is used to tailor the extent of the surgery, as systematic pelvic and para-aortic lymphadenectomy generally is not considered in other than primary ovarian cancers. However, it may not be conclusive, and in some cases, the metastatic nature of the tumor is revealed in the definitive paraffin-embedded sample. Fortunately, immunohistochemistry can address this issue and help distinguish primary ovarian carcinomas from STOs [12].

In general, the histological features favoring metastases include an infiltrative growth pattern with stromal desmoplasia, a nodular growth pattern, involvement of the ovarian surface and superficial cortex, and hilar and lymphovascular space involvement [54].

### The role of immunohistochemistry in histopathological evaluation

Immunohistochemical (IHC) evaluation should always be employed in ovarian tumors in addition to morphology evaluation since it provides additional information [38]. Cytokeratin 7 and 20 (CK7 and CK20) are the most commonly determined antigens in ovarian tumors. Primary ovarian carcinomas are almost always CK7 positive (90–100%), whereas the immunoreactivity to CK20 is generally negative [6]. Other frequently used markers include WT1 and CA 125, which are associated with primary ovarian carcinoma. However, their expression varies in different histologic subtypes: they are positive in most primary ovarian serous adenocarcinomas (both high-grade and low-grade), but are negative in the majority of ovarian clear cell and mucinous carcinomas [56, 57]. The expression of both progesterone receptors (PRs) and estrogen receptors (ERs) in primary ovarian carcinomas is highly variable, depending of the tumor type. In general, ERs are more commonly expressed than PRs and while the expression of ERs and PRs is detected only in about 10% of mucinous and clear cell carcinomas, ERs positivity may be observed in more than 80% of serous and endometrioid carcinomas and the expression of PRs in 30–70% of these tumor types [54]. Noticeably, ERs and PRs also are expressed in breast carcinoma and in other tumors arising from the female genital tract [57, 58]. The immunophenotypes of selected primary and secondary ovarian tumors are listed in Table 2.

**Table 2:** Immunohistochemical profile of primary versus secondary ovarian carcinoma

	Positive	Negative
Primary ovarian carcinoma		
Serous [59]	CK7, CA 125, HAM56, PAX8	CK20
Mucinous [59]	CK7, CK20, MUC5AC_(VE)_, HAM56, CEA, PAX8	CA 125_(VE)_
Endometrioid [57, 59]	CK7, CA 125, HAM54, ER, PR, PAX8	CK20, CEA
Metastatic carcinoma		
Colorectum [59]	CK20, CEA. CDX2	CK7_(VE in mucinous)_, CA 125, MUC5AC, HAM56_(VE)_
Appendix [59]	CK20, MUC5AC_(VE)_, CEA	CK7_(VE)_, CA 125
Stomach [59]	CK7,CK20_(VE)_, MUC5AC	CA 125, HAM56_(VE)_
Breast [57, 60, 61]	GCDFP15, mammaglobin, GATA3, ER_(VE)_, PR_(VE)_	vimentin, WT1, CA 125_(VE)_
Pancreas [57, 59]	CK7, CK20_(VE)_, MUC5AC, CEA, CA 19-9	CA 125, HAM56, DPC4_(negative in ~50%)_
Renal cell carcinoma (clear cell) [62]	vimentin, AE1/AE3, CD10, RCC, PAX8	CK7, CK20, 34βE12
Cervical carcinoma [57]	p16, CEA, HPV infection	ER, PR

*VE* variable expression

The most frequent differential diagnostic question is to distinguish metastatic colorectal cancer (mCRC) from primary ovarian endometrioid or mucinous adenocarcinomas [38]. Primary ovarian endometrioid adenocarcinomas almost always exhibit diffuse positivity for CK7 and CA 125, while CK20, carcinoembryonic antigen (CEA), and CDX2 are usually negative [58, 63]; the converse immunophenotype (CK20^+^, CEA^+^, CDX2^+^, CK7^−^, and CA 125^−^) is observed in mCRC [38]. The distinction between primary ovarian mucinous carcinoma and mucinous mCRC is more difficult, and IHC is less helpful, because primary ovarian mucinous adenocarcinomas often exhibit intestinal differentiation and may be positive for CK20, but also for CEA, CDX2, and CA19-9 [38, 64]; conversely, some mucinous mCRC may be positive for CK7, in which case it is the most useful marker. It is diffusely positive in ovarian carcinoma and negative or only focally positive in mCRC [57]. mCRC with mucinous appearance usually exhibit diffuse CK20 positivity, whereas primary ovarian mucinous carcinomas are usually CK20 negative or only focally positive [38]. Moreover, ovarian mucinous adenocarcinomas are negative for β-catenin and positive for MUC5AC, whereas mCRC exhibits a reverse pattern [57, 65, 66]. CDX2 is a sensitive marker for mCRC, but it is non-specific and can also be expressed in ovarian mucinous carcinoma [67]. A novel marker, dipeptidase 1 (DPEP1), was found to be of clinical use in distinguishing primary mucinous ovarian cancer from ovarian metastasis of GI cancers [68]; if combined with other IHC markers (CDX2 and CK7), together with a single clinical factor (tumor size), DPEP1 can provide an accuracy of 93% [68].

Breast and ovarian cancer are not a rare combination in a patient either with or without an underlying BRCA 1/2 mutation [69]. This often leads to situations when a patient with a history of breast cancer presents with a newly diagnosed adnexal mass, and the origin of the ovarian tumor has to be determined. Gross cystic disease fluid protein-15 (GCDFP-15) and vimentin staining are used most frequently in this setting as breast carcinomas are usually positive for GCDFP-15 and negative for vimentin in most cases, whereas primary ovarian carcinoma is usually negative for GCDFP-15 and often variably positive for vimentin [57]. Other useful markers include mammaglobin and GATA3 (usually positive in breast carcinoma and negative in ovarian carcinoma) [60, 70]. WT1, CA 125, and PAX8 are usually positive in high-grade serous ovarian carcinoma and negative in breast carcinoma; however, in some cases of breast cancer, CA 125 and WT1 also might be positive [61, 71]. To distinguish metastasis of breast and gastric cancer, hepatocyte nuclear factor 4A (HNF4A) seems to be a very potent biomarker (positive in gastric carcinoma and negative in breast carcinoma) [72].

Other specific IHC markers can be used to distinguish metastases from less common primary sites, including renal cell carcinoma (CK7^−^, CD10^+^, RCC^+^) [73], cervical carcinoma (p16^+^, ER^−^, PR, and positive HPV status) [74–76], and malignant melanoma (S-100, MART-1, HMB-45, and SOX10) [77]. It should be emphasized that no antibody is entirely specific nor sensitive for a given neoplasm, and panels of antibodies should be employed when IHC is used to distinguish between various tumor types [38].

### Molecular analysis and its role in the diagnosis of STO

Gene expression profiling is an effective approach in identifying the primary tumor site in CUP [78]. The expression levels of messenger RNA (mRNA) or micro-RNA (miRNA) can be assessed using reverse-transcription polymerase chain reaction (RT-PCR) or oligonucleotide microarray technology [79, 80]. Currently, two commercially available assays capable of identifying the primary tumor in approximately 80% of cases are available [78, 81]. The clinical utility of these assays is supported by the results of a phase II trial, suggesting a survival benefit in patients treated with site-specific therapy based on a gene expression profile [81]; moreover, the results of a phase III trial addressing this issue (GEFCAPI 04) are incoming [82].

Additionally, molecular profiling may aid differential diagnosis between primary ovarian tumors and STOs in case of inconclusive IHC. A study performing comprehensive genomic analysis (a single nucleotide polymorphism [SNP] assay) and transcriptomic analysis to distinguish primary ovarian tumors from metastases from previously diagnosed breast cancer was able to discriminate primary ovarian tumors from breast cancer metastases in concordance with pathological diagnosis and further differentiated four cases of uncertain pathological diagnosis [83]. This study performed a hierarchical clustering of obtained samples combined with a dataset of well-identified primary ovarian tumors and STOs. Interestingly, the genomic and transcriptomic analyses showed complete overleap in the obtained results. Thus, SNP array analysis represents a potent tool to distinguish primary ovarian tumors from STOs. Furthermore, transcriptomic analysis may be used in case the primary tumor tissue specimen is not available [83]. In a previously published study, an array-based comparative genomic hybridization was used to distinguish between primary and metastatic ovarian and endometrial tumors, providing a clear diagnosis in histopathologically equivocal cases [84].

## Treatment

There are no uniform guidelines in the treatment of STOs, as they represent an extremely heterogeneous group of tumors with distinct biological characteristics and prognosis. Moreover, the rarity of these tumors makes any prospective randomized clinical trials practically unfeasible. Therefore, the management of STOs should be based on a thorough diagnostic process to assess the primary tumor site, its biological characteristics, and the extent of the disease. If the primary tumor is detected, it should be treated according to its histological type and stage [42]. There are, however, several intriguing questions regarding STO management, namely the role of cytoreductive surgery and the use of adjuvant chemotherapy after metastasectomy.

### The role of cytoreductive surgery in STO management

While there is clear evidence that surgical cytoreduction in primary ovarian cancer benefits survival, there is little information regarding the role of cytoreductive surgery in STOs. Unfortunately, there are no current prospective trials regarding this issue. Most retrospective studies agreed that cytoreductive surgery may provide a survival benefit in select sub-groups of patients. The primary tumor site seems to be the most relevant factor when considering this approach. Patients with ovarian metastases from CRC appear to be the best candidates because the survival benefit from cytoreductive surgery has been confirmed in most studies [9, 10, 16, 85]. Patients with disease confined to the pelvis have better prognosis than those with extra-pelvic metastases [86]. There is available data indicating that an optimal cytoreduction in patients with mCRC confined to ovaries may result in a higher 5-year survival rate than in those with optimally resected isolated pulmonary or liver metastases [87].

The benefit of cytoreductive surgery in gastric cancer is less clear, and although there are studies reporting the survival benefit from cytoreductive surgery [88–90], results from other studies are rather contradictory [91, 92]. Moreover, STOs of gastric origin have poorer prognosis, compared with other primary tumors, and they are usually associated with lower performance status (PS), anemia, coagulation dysfunction, and cachexia, often preventing the patient from undergoing surgery [85]. There is a difference between synchronous and metachronous STOs of gastric origin in terms of higher feasibility of R0 resection and better survival following resection in metachronous fashion [90]. Taking this into consideration, it seems reasonable to perform a cytoreductive surgery in metachronous STOs of gastric origin confined to ovaries in patients with good PS. In the case of synchronous metastases, the benefit of this approach is less clear, although when combined with hyperthermic intraperitoneal chemotherapy (HIPEC), it might provide a survival benefit with acceptable morbidity and mortality rates [90]. Therefore, the decision whether to perform cytoreductive surgery must be made individually for each patient after considering all of the above mentioned aspects.

The evidence supporting metastasectomy in STOs of breast cancer origin is even less clear. Cytoreductive surgery in breast cancer metastasizing into ovaries did not provide any survival benefit, as observed by Ayhan et al. [10]. There was a trend toward better survival in patients with no visible residual disease after metastasectomy for abdominal/pelvic masses originating from breast cancer compared with those left with gross disease; however, it did not reach any statistical significance [93, 94]. At present, there is not enough evidence supporting the routine use of metastasectomy in STOs of breast origin.

Similar to primary ovarian cancer, the extent of cytoreductive surgery is an important prognostic factor, as it was found that 5-year survival in patients with residual disease less than 2 cm in diameter is significantly higher than in those with residual disease greater than 2 cm in diameter, especially in patients with primary CRC [9, 16]. Furthermore, a significantly longer median OS was observed with no residual disease compared to residual disease less than 0.5 cm [22], indicating that an aggressive debulking surgery may be beneficial and a maximal surgical effort should be made in patients with STOs [9, 95]. Bilateral oophorectomy should be performed during the surgery for ovarian metastasis, even in the case of a tumor confined to a single ovary, as the contralateral ovary can be a site for metachronous metastases, thus preventing the patient from undergoing additional tumor resection [95].

The presence of both ovarian and extraovarian metastases is another factor to be considered, as patients with combined metastases have significantly worse prognosis and an optimal cytoreduction is less frequently feasible [22, 87]. However, patients with extensive mCRC who are able to undergo optimal cytoreduction can experience prolonged disease-free survival. Therefore, the feasibility of optimal cytoreduction should be considered carefully in extra-ovarian mCRC. The PS was identified as another independent prognostic factor of survival [85, 89].

In conclusion, there is a subgroup of patients who may benefit from cytoreductive surgery. These include patients with good PS, metastases limited to ovaries, primary tumor of colorectal origin, and a feasibility of no or minimal residual disease. The data supporting cytoreductive surgery in patients with STOs of gastric origin is less clear, and this approach should be considered on a case-by-case basis. It is worth noting that results of the abovementioned studies must be interpreted with caution, as some of them could be affected by a selection bias, e.g. patients not suitable for cytoreductive surgery had either more extensive disease or other adverse factors preventing them from undergoing surgery. The retrospective nature of these studies and imbalance between resection and non-resection groups are other limiting factors; therefore, further prospective studies are warranted.

### Adjuvant chemotherapy following cytoreductive surgery

There is data indicating that adjuvant chemotherapy following cytoreductive surgery in STOs is able to prolong survival [16, 89, 90]. Platinum-based chemotherapy regimens seem to provide a survival benefit in gastric cancer [89]. Cheong et al. found no difference regarding OS when comparing intravenous and intraperitoneal adjuvant chemotherapy in patients with resected STOs of gastric origin [88]. In contrast to this, Rosa et al. found significantly longer median OS in patients who received a combination of HIPEC and systemic chemotherapy compared with those who received only systemic chemotherapy (33 vs. 20 months, P = 0.0005) [90]. Although the data regarding the role of adjuvant chemotherapy following metastasectomy in STOs of colorectal origin is limited, the experience from patients undergoing resection of lung or liver metastases support the use of adjuvant chemotherapy [96]. Most studies using the combination of 5-fluorouracil (5FU)/leucovorin (LV) observed a benefit in progression-free survival (PFS) although without a statistically significant effect on OS [97, 98]. Triplets containing oxaliplatin seem to provide better results than 5-FU/LV alone; however, the use of triplets containing irinotecan cannot be recommended, as it did not show any benefit over 5-FU/LV in an adjuvant setting following resection of the primary CRC [99]. Although adjuvant chemotherapy following metastasectomy of STOs seems to provide a survival benefit, its use remains controversial considering that there are currently no randomized prospective trials available.

## Conclusion

The metastasis to ovaries from various primary tumor sites is not a rare event, and several possible metastatic pathways, including lymphogenous, hematogenous, and transcoelomic spread have been suggested, highlighting that different primary tumors probably use different preferential metastatic pathways. Secondary tumors of the ovaries may resemble primary ovarian cancer, especially in the case of mucinous adenocarcinomas. Therefore, the potential metastatic nature of all newly diagnosed adnexal masses should always be considered. Further, immunohistochemistry plays a major role in distinguishing primary from secondary ovarian tumors and may suggest the potential primary tumor site. The prognosis of these tumors is generally dismal, although there are differences among distinct histological subtypes. Cytoreductive surgery may provide a survival benefit in select sub-groups of patients, including those patients with good performance status, metastases limited to ovaries, primary tumor of colorectal origin, and feasibility of no or minimal residual disease. The data supporting cytoreductive surgery in patients with secondary ovarian tumors of gastric origin is less clear and this approach should be considered on a case-by-case basis. Although the role of adjuvant chemotherapy following resection of metastases is not clear, it seemingly provides survival benefit in the case of gastric and colorectal cancer. Although the data is limited, platinum-based regimens in gastric cancer and 5-FU/LV, in combination with oxaliplatin in colorectal cancer, appear to improve the clinical outcome. However, prospective randomized trials are warranted to address these issues.
